# Antibiotic-resistant *Escherichia coli* and *Salmonella* spp. associated with dairy cattle and farm environment having public health significance

**DOI:** 10.14202/vetworld.2019.984-993

**Published:** 2019-07-08

**Authors:** Md. Abdus Sobur, Abdullah Al Momen Sabuj, Ripon Sarker, A. M. M. Taufiqur Rahman, S. M. Lutful Kabir, Md. Tanvir Rahman

**Affiliations:** 1 Department of Microbiology and Hygiene, Faculty of Veterinary Science, Bangladesh Agricultural University, Mymensingh 2202, Bangladesh; 2 Adhunik Sadar Hospital, Naogaon, Bangladesh

**Keywords:** one-health, antibiotic resistance genes, dairy farm, *Escherichia coli*, carbapenem resistance, *Salmonella* spp, virulence

## Abstract

**Aim:**

The present study was carried out to determine load of total bacteria, *Escherichia coli* and *Salmonella* spp. in dairy farm and its environmental components. In addition, the antibiogram profile of the isolated bacteria having public health impact was also determined along with identification of virulence and resistance genes by polymerase chain reaction (PCR) under a one-health approach.

**Materials and Methods:**

A total of 240 samples of six types (cow dung – 15, milk – 10, milkers’ hand wash – 10, soil – 10 water – 5, and vegetables – 10) were collected from four dairy farms. For enumeration, the samples were cultured onto plate count agar, eosin methylene blue, and xylose-lysine deoxycholate agar and the isolation and identification of the *E. coli* and *Salmonella* spp. were performed based on morphology, cultural, staining, and biochemical properties followed by PCR.The pathogenic strains of *E. coli* *stx1, stx2*, and *rfbO157* were also identified through PCR. The isolates were subjected to antimicrobial susceptibility test against 12 commonly used antibiotics by disk diffusion method. Detection of antibiotic resistance genes *ereA, tetA, tetB*, and *SHV* were performed by PCR.

**Results:**

The mean total bacterial count, *E. coli* and *Salmonella* spp. count in the samples ranged from 4.54±0.05 to 8.65±0.06, 3.62±0.07 to 7.04±0.48, and 2.52±0.08 to 5.87±0.05 log colony-forming unit/g or ml, respectively. Out of 240 samples, 180 (75%) isolates of *E. coli* and 136 (56.67%) isolates of *Salmonella* spp. were recovered through cultural and molecular tests. Among the 180 *E. coli* isolates, 47 (26.11%) were found positive for the presence of all the three virulent genes, of which *stx1* was the most prevalent (13.33%). Only three isolates were identified as enterohemorrhagic *E. coli*. Antibiotic sensitivity test revealed that both *E. coli* and *Salmonella* spp. were found highly resistant to azithromycin, tetracycline, erythromycin, oxytetracycline, and ertapenem and susceptible to gentamycin, ciprofloxacin, and imipenem. Among the four antibiotic resistance genes, the most observable was *tetA* (80.51-84.74%) in *E. coli* and *Salmonella* spp. and *SHV* genes were the lowest one (22.06-25%).

**Conclusion:**

Dairy farm and their environmental components carry antibiotic-resistant pathogenic *E. coli* and *Salmonella* spp. that are potential threat for human health which requires a one-health approach to combat the threat.

## Introduction

Antibiotic resistance is a global public health concern [1]. Antibiotic-resistant bacteria as the etiology of infection have been expanding at an alarming rate [2]. It is stated that almost 10 million people will die per year due to antimicrobial resistance () infections [3]. At present, drug-resistant microorganisms are broadly circulating in the environmental settings of the earth, and their negative effect has significantly risen in the past few years [4]. Haphazard use of antibiotics and absence of knowledge are the most imperative variables for the rise, selection, and spread of antibiotic-resistant organisms in the environment [5]. If such things happen continuously, it will bring a disaster to human being. At present, many of the antimicrobial agents are utilized in food animal production for controlling diseases and mostly used as growth promoter that is continuously disseminating in human food chain leads serious health problem in human and animals [6]. Cattle in dairy farm could be a potential source for the contamination of the farm environment and farm products by antibiotic-resistant *Escherichia coli* and *Salmonella* spp. present in cow dung. Moreover, these resistance elements can transfer to the people working on the farm directly from contaminated soil, water, and milk to cause serious human health problems [7].

*E. coli* is known as dangerous pathogens in the dairy farm sector worldwide as it causes significant economic losses [8]. There are several strains in *E. coli* and most of them are harmless, but a few of them cause serious foodborne infection in human [9]. Farm animals, especially cattle, asymptomatically carry Shiga toxin-producing *E. coli* (STEC) and enterohemorrhagic *E. coli* (EHEC). These pathogens are zoonotic in nature and can transmit to human from farm through contaminated milk, meat, water, and direct contact with animals or their environmental equipment [10,11]. *Salmonella* spp. is the most ubiquitous organisms in nature and major foodborne zoonotic pathogen, it is also one of the pathogens listed in the WHO priority pathogen list. Dairy cattle act as a reservoir of *Salmonella* spp. that cause salmonellosis in human [12]. *Salmonella* spp. can transmit through feces from infected cattle and their environment. In the past few years, *Salmonella* serotypes have become resistant to frequently used antibiotics that increased the treatment cost in food animal production [13]. Livestock manure contains microbial constituents, which make it a potential source of pathogenic microorganisms for animals and human. About 151.3 million tons of fresh farm animal manure are produced in Bangladesh annually that are mostly used as biofertilizer in agriculture land [14]. Several bacterial pathogens such as *E. coli, Campylobacter, Salmonella, Listeria, Coxiella*, and *Mycobacterium* have been recovered from manure that could be antibiotic-resis­tant and zoonotic in nature [15]. These pathogens can enter into the food chain when manure used as fertilizer in agriculture for crop, vegetables, and fruit production to interferer consumers health [16].

The emergence of antibiotic-resistant bacteria and their resistance genes has turned into a serious growing issue in current medication. There is lack of adequate surveillance data on the occurrence of antibiotic-resistant bacteria in livestock farming system in Bangladesh, especially in dairy cattle and the farm environment focusing one-health.

The present study was therefore designed using a one-health approach to determine the load of total bacteria, *E. coli* and *Salmonella* spp. in dairy cattle and farm environmental components as well as to determine their virulence genes, antibiogram phenotype, and genotype having public health significance.

## Materials and Methods

### Ethical approval and informed consents

No ethical approval was required; however, during the collection of samples; verbal permission was taken from the farm owners and farm workers.

### Study area

The study was conducted on different dairy farms of Mymensingh district of Bangladesh namely, Research Animal farm Bangladesh Agricultural University (BAU), BAU dairy farm, dairy farm of Sutiakhali and Boira. These farms were selected on their use of cow dung as fertilizer for vegetable production. Cow dung, milk, milker’s hand wash, soil, and water were selected for sampling. In addition, vegetables grown in agriculture field within the farm where cow dung used as fertilizer were also collected.

### Sample collection

A total of 240 samples of six items were collected from four dairy farms where each of the farms contributed 60 samples consist of 15 cow dung, 10 milk, 10 milkers’ hand wash, 10 soil, 5 water, and 10 vegetables. All the samples were taken aseptically by utilizing sterile zipper bag. Just after defecation, cow dung samples were collected. Sterile plastic spoon, container, and falcon tube were used for the collection of soil, milk, and water samples, respectively. Milker’s hand wash samples were also collected by washing the hand with phosphate-buffered saline and vegetable samples red spinach (*Amaranthus gangeticus*), Malabar spinach (*Basella alba*), green chili (*Capsicum annum*), and tomato (*Solanum lycopersicum*) from the vegetable production land. After collection, they were transported to the microbiology laboratory, Department of Microbiology and Hygiene, BAU as soon as possible in an ice box. Bacteriological examinations were done promptly before undesirable changes develop.

### Sample processing

Solid (cow dung, soil, and vegetables) and liquid (milk, milker’s hand wash, and water) samples were measured, respectively, in gram and ml. For cow dung and soil samples, 10 g sample and 90 ml 0.1% peptone water were taken in a beaker and mixed well to have the initial dilution. Vegetable samples collected were chopped into small pieces with a sterile knife and mixed homogeneously. A 25 g of these chopped vegetables was taken into a flask containing 225 ml 0.1% peptone water, and vigorously shook to homogenize [17]. Ten ml sample and 90 ml diluent were taken to prepare the initial dilution for a liquid sample. Finally, ten-fold serial dilution was made from all the initial dilutions for the bacterial count.

### Bacteriological analysis

Initially, a ten-fold dilution of the sample was prepared in 0.1% peptone water in Eppendorf tube. Earlier, a plate count agar (PCA) was divided into four parts and marked separately. Four consecutive dilutions within the range of 10^-1^-10^-6^ were taken based on sample types for the four separate parts. Three drops of 10 µl from each dilution were inoculated into each part of the PCA plate separately and incubated at 37°C for 24 h for development of single colonies . After incubation, colonies were counted from three drops of a particular dilution where the average colony count of those three drops was 3-30/10 µl [18]. The results of the total bacterial count were expressed as colony-forming unit (CFU)/g or ml of sample. Similar methods were also applied for counting of *E. coli* and *Salmonella* spp. using eosin methylene blue (EMB) agar and xylose-lysine deoxycholate (XLD) agar, respectively.

### Isolation and identification of bacteria

For obtaining pure culture, bacterial growth on EMB and XLD agar was further streaked on their respective media and incubated overnight at 37°C. Bacteria were identified on the basis of colony characteristics, morphological characteristics by Gram’s staining and biochemical characteristics, namely basic sugar fermentation test, methyl red test, Voges-Proskauer test, and indole test [19]. Final confirmation was done through molecular characterization by polymerase chain reaction (PCR). For PCR, DNA was extracted from pure culture using boiling methods following the procedures of Mahmud *et al*. [20]. PCR for genus-specific *E. coli* and *Salmonella* spp. was performed using previously studied primers (Table-1) following the standard protocol [21,22]. STEC were detected by PCR targeting *stx1* and *stx2* genes, while EHEC detection was based on detection of *rfbO157* gene according to published methods [23,24].

**Table 1:** List of primers used.

Target genes	Primer sequence(5’- 3’)	Approximate band size(bp)	Annealing temperature(°C)	References
*E.coli* 16S rRNA	F: GACCTCGGTTTAGTTCACAGA R: CACACGCTGACGCTGACCA	585	55	[21]
*invA*	F: ATCAGTACCAGTCGTCTTATCTTGAT R: TCTGTTTACCGGGCATACCAT	211	58	[22]
*st×1*	F: ACAATCAGGCGTCGCCAGCGCACTTGCT R: TGTTGCAGGGATCAGTGGTACGGGGATGC	606	58	[23]
*st×2*	F: CCACATCGGTGTCTGTTATTAACCACACC R: GCAGAACTGCTCTGGATGCATCTCTGGTC	372	58	[23]
*rfbO157*	F: AAGATTGCGCTGAAGCCTTTG R: CATTGGCATCGTGTGGACAG	497	66	[24]
*ereA*	F: GCCGGTGCTCATGAACTTGAG R: CGACTCTATTCGATCAGAGGC	419	52	[27]
*tetA*	F: GGTTCACTCGAACGACGTCA R: CTGTCCGACAAGTTGCATGA	577	57	[28]
*tetB*	F: CCTCAGCTTCTCAACGCGTG R: GCACCTTGCTGATGACTCTT	634	56	[28]
*SHV*	F: TCGCCTGTGTATTATCTCCC R: CGCAGATAAATCACCACAATG	768	52	[27]

E. coli=Escherichia coli

### Antimicrobial susceptibility test

All the isolated *Salmonella* spp. and *E. coli* were subjected to antimicrobial susceptibility test against 12 commonly used antibiotics, i.e., azithromycin (15 µg), chloramphenicol (30 µg), ciprofloxacin (5 µg), erythromycin (15 µg), gentamycin (10 µg), kanamycin (30 µg), neomycin (30 µg), oxytetracycline (30 µg), ertapenem (10 µg), meropenem (10 µg), imipenem (10 µg), and tetracycline (30 µg) following the disk diffusion methods described by Bauer *et al*. [25]. Finally, the zone of growth inhibition was compared with standards provided by the Clinical and Laboratory Standards Institute [26] to identify the resistant isolates.

### Molecular detection of antibiotic resistance genes

Isolates of *E. coli* and *Salmonella* spp. that showed resistance to erythromycin, tetracycline, and beta-lactam (ertapenem, meropenem, and imipenem) phenotypically were further screened for the detection of *ereA, tetA*, and *tetB* and *SHV* resistance genes, respectively. The primers used for the detection of resistance genes are listed in Table-1 [27,28].

### Statistical analysis

SPSS software version 20.0 (IBM, USA) was used to analyze the data. Frequency and mean were estimated using descriptive analysis.

## Results

### Microbial load

The microbial analysis revealed that the average total viable bacterial count (TVC) ranged from 4.54±0.05 to 8.65±0.06 log CFU/g or mlmean±standard deviation (SD) among all the samples analyzed. The highest TVC was observed in soil and lowest in water samples of Sutiakhali dairy farm. The highest *E. coli* count was found as 7.04±0.48 log CFU/g±SD in cow dung samples of Boira dairy farm and lowest as 3.62±0.07 log CFU/ml±SD in water samples of Sutiakhali dairy farm. The highest *Salmonella* spp. count was found 5.87±0.05 log CFU/g±SD and lowest 2.52±0.08 log CFU/ml±SD in soil and water samples of Sutiakhali dairy farm, respectively (Table-2).

**Table 2:** Mean total bacteria, *E.coli* and *Salmonella* spp. counts in dairy cattle and farm environmental samples (log CFU/g or ml±SD).

Sample(n)	Research animal farm			BAU dairy farm			Dairy farm in Sutiakhali			Dairy farm in Boira		
			
	TVC	TEC	TSC	TVC	TEC	TSC	TVC	TEC	TSC	TVC	TEC	TSC
Cow dung(15)	7.93±0.044	6.98±0.041	5.74±0.066	7.96±0.047	7.00±0.042	5.78±0.069	7.99±0.045	7.03±0.042	5.83±0.062	7.99±0.054	7.04±0.048	5.82±0.084
Milk(10)	6.76±0.039	4.88±0.028	3.76±0.051	6.79±0.038	4.90±0.029	3.79±0.049	6.83±0.032	4.93±0.023	3.84±0.039	6.88±0.036	4.97±0.033	3.87±0.036
Milker’s hand wash(10)	6.57±0.067	5.75±0.047	4.58±0.060	6.63±0.062	5.80±0.042	4.64±0.083	6.68±0.052	5.83±0.044	4.69±0.061	6.71±0.044	5.85±0.040	4.71±0.057
Soil(10)	8.56±0.065	6.66±0.058	5.81±0.038	8.62±0.080	6.71±0.078	5.85±0.043	8.65±0.059	6.74±0.051	5.87±0.050	8.64±0.071	6.73±0.07	5.87±0.051
Water(5)	4.70±0.089	3.80±0.101	2.74±0.116	4.65±0.067	3.74±0.068	2.69±0.084	4.54±0.047	3.62±0.067	2.52±0.083	4.58±0.080	3.66±0.080	2.56±0.063
Vegetables(10)	6.84±0.045	5.57±0.087	4.57±0.091	6.87±0.047	5.59±0.101	4.63±0.091	6.87±0.044	5.53±0.048	4.63±0.064	6.91±0.039	5.60±0.095	4.68±0.076

TVC=Total viable bacterial count, TEC=Total *E.coli* count, TSC=Total *Salmonella* spp. count, CFU=Colony-forming unit, SD-Standard deviation, BAU=Bangladesh Agricultural University, *E.coli*=*Escherichia* *coli*

### Isolation and identification of *E. coli* and *Salmonella* spp.

**Figure-1:**
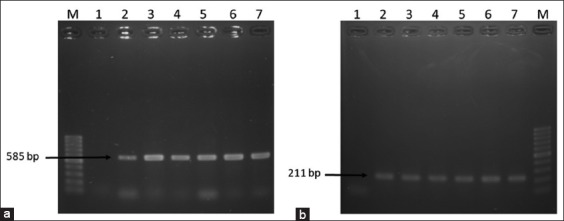
Polymerase chain reaction (PCR) amplification of 16S rRNA of *Escherichia coli* and *invA* gene of *Salmonella* spp. (a) PCR amplification of 16S rRNA of *E. coli*. Lane M: 100 bp DNA Marker, 1: Negative control, 2: Positive control, and 3-7: Representative *E. coli* isolates. (b) PCR amplification of *invA* gene of *Salmonella* spp. Lane M: 100 bp DNA Marker, 1: Negative control, 2-6: Representative *Salmonella* spp. isolates, and 7: Positive control.

A total of 180 (75%) *E. coli* and 136 (56.67%) *Salmonella* spp. were isolated from the 240 samples through cultural and molecular tests (Figure-1). Among these, the highest *E. coli* (92.5%) and *Salmonella* spp. (72.5%) were detected in soil samples and lowest in water samples (Table-3).

**Table 3:** Prevalence of *E.coli* and *Salmonella* spp. in dairy cattle and farm environment.

Source of sample	Cow dung(%)		Milk(%)		Milker’s hand wash(%)		Soil(%)		Water(%)		Vegetables(%)	
					
	*E.coli*	*Salmonella* spp.	*E.coli*	*Salmonella* spp.	*E.coli*	*Salmonella* spp.	*E.coli*	*Salmonella* spp.	*E.coli*	*Salmonella* spp.	*E.coli*	*Salmonella* spp.
RAF	11/15 (73.33)	9/15 (60)	6/10 (60)	4/10 (40)	7/10 (70)	5/10 (50)	8/10 (80)	6/10 (60)	2/5 (40)	1/5 (20)	7/10 (70)	5/10 (50)
BDF	11/15 (73.33)	10/15 (66.67)	4/10 (40)	3/10 (30)	6/10 (60)	4/10 (40)	9/10 (90)	6/10 (60)	1/5 (20)	1/5 (20)	7/10 (70)	4/10 (40)
DFS	12/15 (80)	10/15 (66.67)	8/10 (80)	5/10 (5)	8/10 (80)	6/10 (60)	10/10 (100)	8/10 (80)	3/5 (60)	2/5 (40)	8/10 (80)	5/10 (50)
DFB	13/15 (86.67)	13/15 (86.67)	9/10 (90)	6/10 (60)	8/10 (80)	7/10 (70)	10/10 (100)	9/10 (90)	3/5 (60)	2/5 (40)	9/10 (90)	5/10 (50)
Total	47/60 (78.33)	42/60 (70)	27/40 (67.5)	18/40 (45)	29/40 (72.5)	22/40 (55)	37/40 (92.5)	29/40 (72.5)	9/20 (45)	6/20 (30)	31/40 (77.5)	19/40 (47.5)

RAF=Research animal farm, BDF=BAU dairy farm, DFS=Dairy farm in Sutiakhali, DFB=Dairy farm in Boira, *E.coli*=*Escherichia* *coli*, BAU=Bangladesh Agricultural University

### Determination of virulent genes of *E. coli*

**Figure-2:**
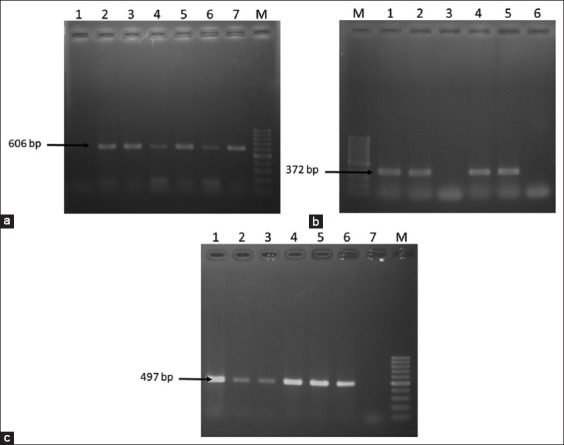
Polymerase chain reaction (PCR) amplification of virulence genes of *Escherichia coli*. (a) PCR amplification of *stx1*gene of *E. coli*. Lane M: 100 bp DNA Marker, 1: Negative control, 2-6: Representative *E. coli* isolates, and 7: Positive control. (b) PCR amplification of *stx2* gene of *E. coli*. Lane M: 100 bp DNA Marker, 1: Positive control, 2-5: Representative *E. coli* isolates, and 6: Negative control. (c) PCR amplification of *rfbO157* gene of *E. coli*. Lane M: 100 bp DNA Marker, 1-5: Representative *E. coli* isolates, 6: Positive control, and 7: Negative control.

**Figure-3:**
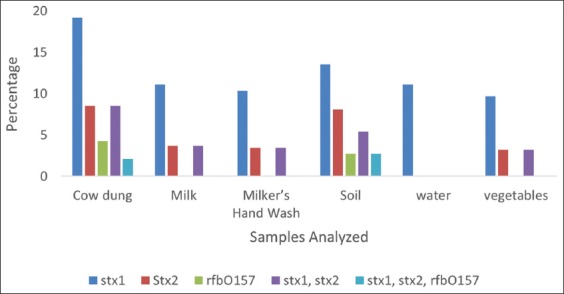
Distribution of *stx1, stx2*, and *rfbO157* in isolated *Escherichia coli*.

Among the 180 *E. coli* isolates, 47 (26.11%) were found positive for the presence of either one or all the three virulent genes (Figure-2), of which *stx1* was the most prevalent (13.33%). Few isolates were also found positive for two of the virulence genes. About 4.44% isolates were found positive for both the *stx1* and *stx2* while two isolates (1.11%) were found to be positive for all the three virulent genes. Only three isolates (1.67%) were identified as EHEC based on the detection of *rfbO157* gene. Among the six types of samples, cow dung was more contaminated with pathogenic *E. coli* strains than other collected samples. Distributions of virulent genes in the isolated *E. coli* in different samples are presented in Figure-3.

### Antimicrobial susceptibility test

Isolated *E. coli* and *Salmonella* spp. were subjected to antimicrobial susceptibility test against 12 commonly used antibiotics including three beta-lactam antibiotics, namely ertapenem, meropenem, and imipenem. From Table-4, it is evident that all the isolates of *E. coli* and *Salmonella* spp. were 100% resistance to azithromycin. *E. coli* was also found highly resistant to tetracycline (89.44%), erythromycin (88.89%), oxytetracycline (78.89%), and ertapenem (66.67%). Similarly, *Salmonella* spp. was found resistant to erythromycin (87.5%), followed by tetracycline (86.76%), oxytetracycline (75.73%), and ertapenem (50%) antibiotics. Both the isolates were highly susceptible to gentamycin, ciprofloxacin, and imipenem.

**Table 4:** Antibiogram of the isolated *E.coli* and *Salmonella* spp.

Antibiotic	*E.coli*							*Salmonella* spp.						
	
	Cow dung(47)	Milk(27)	Milker’s Hand Wash(29)	Soil(37)	Water (9)	Vegetables (31)	Total (180)	Cow dung(42)	Milk (18)	Milker’s Hand Wash (22)	Soil(29)	Water(6)	Vegetables (19)	Total (136)
AZM	47 (100)	27 (100)	29 (100)	37 (100)	9 (100)	31 (100)	180 (100)	42 (100)	18 (100)	22 (100)	29 (100)	6 (100)	19 (100)	136 (100)
C	19 (40.42)	8 (29.63)	9 (31.03)	13 (35.13)	3 (33.3)	9 (29.03)	61 (33.89)	15 (35.71)	5 (27.78)	7 (31.82)	9 (31.03)	2 (33.33)	5 (26.31)	43 (31.62)
CIP	10 (21.28)	4 (14.81)	4 (13.79)	6 (16.22)	1 (11.11)	4 (12.90)	29 (16.11)	8 (19.05)	2 (11.11)	3 (13.64)	4 (13.79)	0 (0)	2 (10.53)	19 (13.97)
E	44 (93.62)	24 (88.89)	25 (86.21)	33 (89.19)	8 (88.89)	26 (83.87)	160 (88.89)	39 (92.86)	15 (83.33)	19 (86.36)	26 (89.65)	5 (83.33)	15 (78.95)	119 (87.5
GEN	6 (12.76)	2 (7.41)	2 (6.90)	4 (10.81)	0 (0)	2 (6.45)	16 (8.89)	4 (9.52)	1 (5.55)	1 (4.54)	2 (6.90)	0 (0)	1 (5.26)	9 (6.62)
K	20 (42.55)	9 (33.33)	9 (31.03)	13 (35.13)	2 (22.22)	6 (19.35)	59 (32.78)	16 (38.09)	5 (27.78)	6 (27.27)	9 (31.03)	1 (16.67)	2 (10.53)	39 (28.68)
N	21 (44.68)	10 (37.04)	9 (31.03)	13 (35.13)	2 (22.22)	6 (19.35)	61 (33.89)	18 (42.86)	6 (33.33)	7 (31.82)	11 (37.93)	2 (33.33)	3 (15.79)	47 (34.56)
O	40 (85.11)	21 (77.78)	22 (75.86)	30 (81.08)	7 (77.78)	22 (70.97)	142 (78.89)	35 (83.33)	13 (72.22)	15 (68.18)	22 (75.86)	4 (66.67)	14 (73.68)	103 (75.73)
ETP	35 (74.47)	18 (66.67)	17 (58.62)	26 (70.27)	6 (66.67)	18 (58.06)	120 (66.67)	25 (59.52)	9 (50)	10 (45.45)	16 (55.17)	2 (33.3)	6 (31.58)	68 (50)
MEM	16 (34.04)	7 (25.92)	7 (24.14)	10 (27.03)	2 (22.22)	7 (22.58)	49 (27.22)	11 (26.19)	4 (22.22)	5 (22.73)	7 (24.14)	1 (16.67)	3 (15.79)	31 (22.79)
IPM	11 (23.40)	5 (18.52)	5 (17.24)	8 (21.62)	1 (11.11)	4 (12.90)	34 (18.89)	7 (16.67)	2 (11.11)	3 (13.64)	4 (13.79)	1 (16.67)	1 (5.26)	18 (13.23)
TE	45 (95.74)	24 (88.89)	24 (82.76)	34 (91.89)	8 (88.89)	26 (83.87)	161 (89.44)	39 (92.86)	15 (83.33)	18 (81.82)	25 (86.21)	5 (83.33)	16 (84.21)	118 (86.76)

AZM=Azithromycin, C=Chloramphenicol, CIP=Ciprofloxacin, E=Erythromycin, GEN=Gentamycin, K=Kanamycin, N=Neomycin, O=Oxytetracycline, ETP=Ertapenem, MEM=Meropenem, IPM=Imipenem, TE=Tetracycline, *E.coli*=*Escherichia* *coli*

### Molecular detection of antibiotic resistance genes

**Figure-4:**
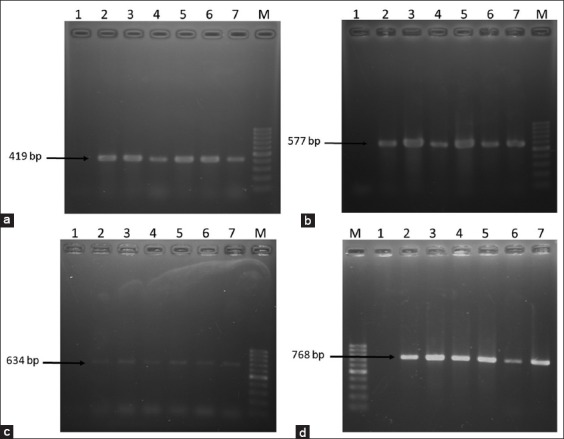
Polymerase chain reaction (PCR) amplification of antibiotic resistance genes of *Escherichia coli* and *Salmonella* spp. (a) PCR amplification of *ereA* gene of erythromycin resistant *E. coli* and *Salmonella* spp. Lane M: 100 bp DNA Marker, 1: Negative control, 2-3: Representative *E. coli* isolates, 4-5: Representative *Salmonella* spp. isolates, 6: Positive control for *E. coli*, and 7: Positive control for *Salmonella* spp. (b) PCR amplification of *tetA* gene of tetracycline resistant *E. coli* and *Salmonella* spp. Lane M: 100 bp DNA Marker, 1: Negative control, 2-3: Representative *E. coli* isolates, 4-5: Representative *Salmonella* spp. isolates, 6: Positive control for *E. coli*, and 7: Positive control for *Salmonella* spp. (c) PCR amplification of *tetB* gene of tetracycline resistant *E. coli* and *Salmonella* spp. Lane M: 100 bp DNA Marker, 1: Negative control, 2-3: Representative *E. coli* isolates, 4-5: Representative *Salmonella* spp. isolates, 6: Positive control for *E. coli*. and 7: Positive control for *Salmonella* spp. (d) PCR amplification of *SHV* gene of ertapenem, imipenem and meropenem resistant *E. coli* and *Salmonella* spp. Lane M: 100 bp DNA Marker, 1: Negative control,2: Positive control for *E. coli*, 3: Positive control for *Salmonella* spp., 4-5: Representative *E. coli* isolates, and 6-7: Representative *Salmonella* spp. isolates.

*E. coli* and *Salmonella* spp. that showed phenotypically resistance to erythromycin, tetracycline, and beta-lactam antibiotics were further screened for the detection of *ereA, tetA*, and *tetB* and *SHV* resistance genes (Figure-4). From Table-5, it is evident that *tetA* was the most prevalent resistance genes (80.51-84.47%%) among the four resistance genes both in *E. coli* and *Salmonella* spp. and *SHV* genes were the lowest one (22.06-25%). On a sample basis, bacteria isolated from cow dung harbored the highest resistance genes (50-53.25%) compared to other samples originated from the dairy farms.

**Table 5:** Distribution of antibiotic resistance genes of the isolated *E.coli* and *Salmonella* spp.

Resistance gene	*E.coli*						

	Cow dung	Milk	Milker’s hand wash	Soil	Water	Vegetables	Total
*ereA*	19/44 (43.18)	8/24 (33.33)	8/25 (32.00)	12/33 (36.36)	3/8 (37.5)	8/26 (30.77)	58/160 (36.25)
*tetA*	40/45 (88.89)	20/24 (83.33)	21/24 (87.5)	28/34 (82.35)	6/8 (75)	21/26 (80.77)	136/161 (84.47)
*tetB*	21/45 (46.67)	9/24 (37.5)	8/24 (33.33)	13/34 (38.23)	3/8 (37.5)	10/26 (38.46)	64/161 (39.75)
*SHV*	10/35 (28.57)	4/18 (22.22)	4/17 (23.53)	7/26 (26.92)	0/6 (0)	5/18 (27.78)	30/120 (25.00)
Total	90/169 (53.25)	41.90 (45.55)	41/90 (45.55)	60/127 (47.24)	12/30 (40)	44/96 (45.83)	288/603 (47.76)

**Resistance gene**	***Salmonella* spp.**						

	**Cow dung**	**Milk**	**Milker’s hand wash**	**Soil**	**Water**	**Vegetables**	**Total**

*ereA*	16/39 (41.02)	5/15 (33.33)	6/19 (31.58)	9/26 (34.61)	0/5 (0)	4/15 (26.67)	40/119 (33.61)
*tetA*	33/39 (84.61)	12/15 (80.00)	14/18 (77.78)	20/25 (80.00)	4/5 (80.00)	12/16 (75.00)	95/118 (80.51)
*tetB*	16/39 (41.02)	5/15 (33.33)	7/18 (38.89)	9/25 (36.00)	1/5 (20.00)	5/16 (31.25)	43/118 (36.44)
*SHV*	6/25 (24.00)	2/9 (22.22)	2/10 (20.00)	4/16 (25.00)	0/2 (0)	1/6 (16.67)	15/68 (22.06)
Total	71/142 (50)	24/54 (44.44)	29/65 (44.61)	42/92 (45.65)	5/17 (29.41)	22/53 (41.51)	193/423 (45.63)

*E.coli*=*Escherichia* *coli*

## Discussion

Antimicrobial agents are indiscriminately used in animal production system for disease prevention and control resulting in the development of resistance against these agents, particularly in zoonotic bacteria that can easily transfer to human through food chains [29]. Zoonotic antibiotic-resistant microorganisms such as *E. coli* and *Salmonella* spp. now have become the global issue as these organisms may bargain the capacity of different treatment regimens to address sickness and disease in human therapeutic settings [30]. In Bangladesh, not enough baseline data are available on the occurrence of antibiotic-resistant bacteria in dairy farm and farm environment to support the National Action Plan of the Government on AMR. To the best of our knowledge this is the first one-health based comprehensive research on the investigation of antibiotic-resistant *E. coli* and *Salmonella* spp. from dairy cattle, dairy farm environment and farm workers in Bangladesh having public health significance.

Cattle in the farm are continuously shedding cow dung and urine into the soil. Thus, soli of the dairy farm is getting heavily contaminated with bacteria of cow dung and urine. Similarly, the present study showed that among all the samples, the highest TVC was found in soil samples of Sutiakhali dairy Farm (Table-2). Since *E. coli* and *Salmonella* spp. are the part of natural intestinal flora, they were often found in higher number in cow dung. Cow dung contains huge microbial population including pathogenic bacteria that have a potential effect on human and animal health [31]. Contaminated drinking water is the common source of coliform bacteria in dairy farm [32]. In comparison to other collected samples, water samples were found less contaminated by *E. coli*. Hassan *et al*. [33] found the geometric mean of heterotrophic plate count of tap water from Mymensingh, Gazipur, and Sherpur district were 8.4×10^5^, 2.5×10^6^, and 6.8×10^5^ CFU/100 ml that were higher than our study. Milk is the most important output of dairy farm for human. Here, we found the milk samples contaminated with *E. coli* and *Salmonella* spp. This contamination may be due to improper hygiene practice in dairy farm especially hygiene of milker’s hand as higher TVC, *E. coli*, and *Salmonella* spp. count were found in milker’s hand wash than milk sample. Khan *et al*. [34] also investigated milk sample of Boira area and found the TVC and TCC as 5.93 and 2.52 log CFU/ml, respectively, a slightly lower than our findings.

Detection of *E. coli* and *Salmonella* spp. in the samples analyzed originating from various farms was not unexpected, since *E. coli* and *Salmonella* spp. are ubiquitous in nature. In this study, we did not identify the isolated *Salmonella* at species level, but their presence is alarming. However, detail study on identification of these *Salmonella* at species level in underway in another study. They are the part of intestinal microflora of animals and birds. Barlow *et al*. [30], Rodriguez-Rivera *et al*. [35], Navajas-Benito *et al*. [36], Jajarmi *et al*. [37], and Batabyal *et al*. [38] also reported the presence of *E. coli* and *Salmonella* spp. in dairy farm samples. The occurrence of higher prevalence of *E. coli* and *Salmonella* spp. in the dairy farm may be due to improper management of cow dung resulting transmission of *E. coli* and *Salmonella* spp. into dairy farm environment especially milk and water [39]. Vegetable samples analyzed were also found positive for *E. coli* and *Salmonella* spp. This may be linked with the use of untreated cow dung as the organic fertilizer for vegetable production in nearby agriculture land. Mukherjee *et al*. [40] reported that fresh cow dung or improper treatment of cow dung may be the cause of transmission of *E. coli* and *Salmonella* spp. to vegetables on farm.

STEC and EHEC are major human pathogen associated with foodborne illness [37]. In this study, STEC and EHEC were detected from various samples (Figure-3). The prevalence of these pathogens was highest in cow dung. Pathogenic *E. coli* are commonly found in animal feces but their prevalence in current study was quite higher than the previous study. In Bangladesh a study reported, the occurrence of 10% STEC [41] in the cattle fecal samples and in India it was reported as 19% [42]. The observed variations in the occurrence of STEC and EHEC among these studies might be due to different geographic locations and variations in farm management.

Antibiotic resistance is a serious health issue globally. This study focused on distribution and occurrence of antibiotic-resistant *E. coli* and *Salmonella* spp. in dairy farm environment having public health importance. Twelve commonly used antibiotics including carbapenem group were tested in the antibiogram study. All the *E. coli* and *Salmonella* spp. were found 100% resistant to azithromycin. Earlier Islam *et al*. [43] observed lower resistance against azithromycin in *E. coli* isolated from milk in Bangladesh. In the present study, gentamycin, ciprofloxacin, imipenem, meropenem, kanamycin, chloramphenicol, and neomycin were found most effective whereas erythromycin, tetracycline, and ertapenem were found less effective against *E. coli* and *Salmonella* spp. These observed variations in the sensitivity pattern may be linked with the variations of concentration and frequency of the use of these antibiotics in Bangladesh that need further investigation.

Carbapenem group of antibiotics (ertapenem, imipenem, and meropenem) is major choice of antibiotic for treating disease caused by multidrug-resistant *E. coli* infections in human. In veterinary practice, these drugs are not commonly used in Bangladesh yet. Islam *et al*. [44] found imipenem sensitive *E. coli* isolates in clinical samples of human origin in Bangladesh. On the other hand, Mamun *et al*. [45] found *E. coli* isolated from rectal swab of healthy cattle as highly sensitive to carbapenem group of antibiotics study. For the 1^st^ time in Bangladesh, the present study showed that *E. coli* and *Salmonella* spp. isolated from dairy farm are resistant to carbapenem group of antibiotics, e.g. ertapenem (50-66.67%), meropenem (up to 27.79%) and imipenem (13.23-18.89%). Carbapenem-resistant Enterobacteriaceae are listed as the critical group of priority pathogen as defined by the WHO [46].

This may be due to the transmission of carbapenem resistance bacteria from human particularly farm workers and visitors to dairy cattle and farm environment.

Among the resistance genes, *tetA* and *tetB* responsible for resistance against tetracycline were found as the most prevalent. However, this was not unexpected, since tetracycline is one of the most widely used antibiotics in Bangladesh. Previously, Navajas-Benito *et al*. [37] also reported the prevalence of a higher number of tetracycline resistance genes (11/15) in *E. coli* associated with dairy farm. On the other hand, the prevalence of *SHV* and *ereA* genes was found comparatively low in this study as reported elsewhere [47,48].

## Conclusion

The present study identified the widespread occurrence of antibiotic-resistant *E. coli* and *Salmonella* spp. in dairy cattle and farm environment. Many of these isolates were also found pathogenic in nature. From farm, these resistant pathogens can transmit to human through the food chain (contaminated milk and vegetables) or through direct and indirect contact. Practice of good farm management including hygiene and manure treatment need to be established to reduce the chance of transmission of antibiotic-resistant bacteria to human along with judicial use of antibiotics in the dairy cattle. In addition, one-health approaches are need to be adopted to control AMR in humans, animals and environment.

## Authors’ Contributions

MTR, MAS, and AMMTR designed the study. MAS, AAMS, and RS did laboratory work assisted by MTR. AMMTR, and MAS wrote the manuscript and analyzed. MTR, MBR and SMLK critically checked and improved the manuscript. All authors read and approved the final manuscript.
